# Calibration trial of an innovative medical device (*NEVVA^©^*) for the evaluation of pain in non-communicating patients in the intensive care unit

**DOI:** 10.3389/fmed.2024.1309720

**Published:** 2024-06-27

**Authors:** Mathieu Bellal, Julien Lelandais, Thomas Chabin, Aurélie Heudron, Thomas Gourmelon, Pierrick Bauduin, Pierre Cuchet, Cédric Daubin, Célia De Carvalho Ribeiro, Augustin Delcampe, Suzanne Goursaud, Aurélie Joret, Martin Mombrun, Xavier Valette, Damiano Cerasuolo, Rémy Morello, Patrick Mordel, Fabien Chaillot, Jean Jacques Dutheil, Denis Vivien, Damien Du Cheyron

**Affiliations:** ^1^Department of Medical Intensive Care, Caen University Hospital, Caen, France; ^2^Normandie Univ., UNICAEN, INSERM UMRS U1237 PhIND, Caen, France; ^3^Samdoc Medical Technologies Company, Caen, France; ^4^Department of Methodology and Statistics, Caen University Hospital, Caen, France; ^5^Department of Clinical Research, Caen University Hospital, Caen, France; ^6^Department of Biological Resources Center, Caen University Hospital, Caen, France

**Keywords:** pain, facial expression, Behavioral Pain Scale, critically ill patients, artificial intelligence

## Abstract

**Background:**

Pain management is an essential and complex issue for non-communicative patients undergoing sedation in the intensive care unit (ICU). The Behavioral Pain Scale (BPS), although not perfect for assessing behavioral pain, is the gold standard based partly on clinical facial expression. *NEVVA^©^*, an automatic pain assessment tool based on facial expressions in critically ill patients, is a much-needed innovative medical device.

**Methods:**

In this prospective pilot study, we recorded the facial expressions of critically ill patients in the medical ICU of Caen University Hospital using the iPhone and Smart Motion Tracking System (SMTS) software with the Facial Action Coding System (FACS) to measure human facial expressions metrically during sedation weaning. Analyses were recorded continuously, and BPS scores were collected hourly over two 8 h periods per day for 3 consecutive days. For this first stage, calibration of the innovative *NEVVA^©^* medical device algorithm was obtained by comparison with the reference pain scale (BPS).

**Results:**

Thirty participants were enrolled between March and July 2022. To assess the acute severity of illness, the Sequential Organ Failure Assessment (SOFA) and the Simplified Acute Physiology Score (SAPS II) were recorded on ICU admission and were 9 and 47, respectively. All participants had deep sedation, assessed by a Richmond Agitation and Sedation scale (RASS) score of less than or equal to −4 at the time of inclusion. One thousand and six BPS recordings were obtained, and 130 recordings were retained for final calibration: 108 BPS recordings corresponding to the absence of pain and 22 BPS recordings corresponding to the presence of pain. Due to the small size of the dataset, a leave-one-subject-out cross-validation (LOSO-CV) strategy was performed, and the training results obtained the receiver operating characteristic (ROC) curve with an area under the curve (AUC) of 0.792. This model has a sensitivity of 81.8% and a specificity of 72.2%.

**Conclusion:**

This pilot study calibrated the *NEVVA^©^* medical device and showed the feasibility of continuous facial expression analysis for pain monitoring in ICU patients. The next step will be to correlate this device with the BPS scale.

## Graphical Abstract

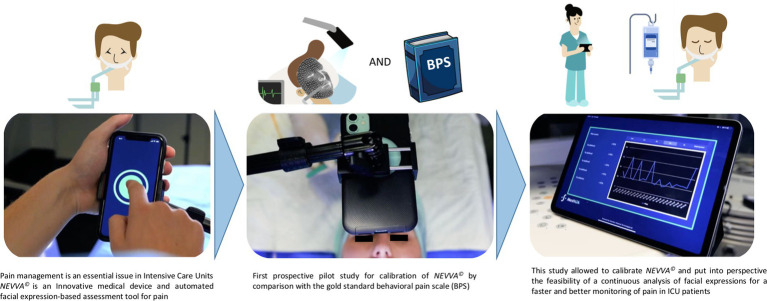

## Highlights

- What is already known: Early pain management in ICUs is one of the cornerstones of standard care in critically ill patients. It is difficult to ensure reliable pain assessment for non-communicative ICU patients. International clinical practice guidelines recommend systematic pain assessment using subjective behavioral scales.
- What this paper adds: Better pain control is necessary in ICUs. Consequently, this calls for the development of new automated pain assessment tools, such as the novel *NEVVA^©^* medical device, based on automated, continuous analysis of facial expressions.

## 1 Introduction

Pain is defined as an unpleasant subjective sensory and emotional experience related to or resembling that associated with actual or potential tissue damage. Nociception is a distinct concept that refers to the physiological neural process of encoding harmful stimuli, which can lead to pain. The effects of encoding noxious stimuli may manifest as autonomic responses (e.g., fluctuations in vital signs) and behavioral responses (e.g., facial expressions) (1). Consequently, these responses can serve as indicators for pain assessment in cases where communication ability is compromised.

Pain is reported in more than 50% of cases in critically ill patients, with physiological and psychological consequences (increased morbidity and mortality, disturbances of the nychthemeral rhythm, post-traumatic stress syndromes, etc.) (2). Pain is associated with different procedures such as surgical incisions, chest tubes, arterial blood sampling, or endotracheal suctioning, and is reported as severe in more than 15% of patients during their stay in the intensive care unit (ICU) (3). Fifty-five percent of ICU nurses, however, underestimate patient pain when asked to rate pain intensity using a visual analog scale (4).

Early ICU pain management is one of the cornerstones of standard care in critically ill patients. Excessive use of inappropriate sedation-analgesia can cause major side effects (alveolar hypoventilation, renal dysfunction, digestive paresis, etc.) and must be avoided. It is necessary to distinguish between an early phase of deep and multimodal sedation-analgesia over the first few hours or days and a later phase of gradual weaning from sedation-analgesia. Deep sedation is defined by a score of −4/−5 on the Richmond Agitation and Sedation scale (RASS), followed by a gradual release of sedation, which is defined by a RASS score of −3 to 0 (Table A1) (5).

The assessment and management of pain in communicating with patients has been the subject of extensive literature (6). Nevertheless, it is difficult to ensure sensitive and reliable pain assessment in the ICU for non-communicative patients under deep multimodal sedation. Behaviors may be masked in heavily sedated patients, in those receiving neuromuscular blocking agents, or in those with severe neurological lesions significantly affecting their motor system. Historical scales rely on hetero-assessment and use physiological variables in response to nociceptive action such as heart rate, respiratory rate, blood pressure, pupil diameter, and sweating without good specificity (7). In adult ICU patients, vital signs are not recommended for pain assessment, and international clinical practice guidelines recommend a systematic pain assessment using subjective behavioral scales, including behavioral indicators of pain strongly correlated with hetero-assessment of pain intensity (8).

Two scales, the Behavioral Pain Scale (BPS) (9) and the Critical Care Pain Observation Tool (CPOT) (10), are commonly used in the ICU. The BPS has three analysis criteria: facial expression, upper limb tone, and compliance with mechanical ventilation (the BPS score is defined between 3 and 12, with each indicator ranging from 1 to 4 in proportion to pain intensity, adapted from the COMFORT and Harris scales in pediatric ICUs) (11) (Table A2). Nevertheless, due to a lack of reproducibility, sensitivity, and specificity, and insufficient discrimination, scientific literature did not allow us to recommend the use of a particular scale (12, 13). Thus, no behavioral scale is considered optimal for non-communicative critically ill patients (14). The sensitive, systematic, discriminatory, and reproducible assessment of pain in ICU therefore remains a challenge for clinicians in order to choose the finest and most appropriate dose of analgesia (15, 16).

In a recent study, Nuseir et al. (17) noted that pain management is multifactorial and complex and could benefit from automated approaches to improve the quality of care. Some automated tools for the recognition of facial expressions of pain have been recently developed using distinct approaches (11–15). However, the use of facial images of ICU patients is not easy in routine clinical practice due to difficulties in obtaining standardized, unmasked facial images (e.g., endotracheal tube, nasoesophageal tube, and oxygen mask). In addition, facial muscle movements associated with pain may be weak due to sedation and tissue edema (e.g., neuromuscular blocking agents or edema-induced loss of dynamic change in the face). In recent years, there has been growing interest in integrating artificial intelligence (AI) into medicine, encompassing various techniques such as machine learning, deep learning, data mining, and natural language processing (18). The literature has recognized the crucial role of AI in clinical settings, particularly in disease diagnosis, treatment selection, and patient monitoring. Applications of AI in pain research have been relatively understudied. Nevertheless, recent advances in AI have enabled us to develop a pain assessment tool based on facial expressions in critically ill patients (19). The AI gold standard for objective assessment of facial expressions in human emotion research is the Facial Action Coding System (FACS) (20). This system measures the individual movements or “Action Units” (AUs), among facial muscles, assigning codes to the activity of individual muscles or muscle groups.

To our knowledge, few studies have focused on AI applications in pain assessment in critically ill, non-communicative ICU patients. In this prospective, pilot study, we recorded the facial expressions of critically ill patients in the medical ICU at Caen University Hospital, using FACS and Smart Motion Tracking System (SMTS) software to build a database and calibrate the innovative medical device—*NEVVA^©^*—on facial expressions.

## 2 Materials and methods

### 2.1 Study population

We conducted a prospective study by enrolling 30 patients who were admitted to the medical ICU at Caen University Hospital in France between March 2022 and June 2022. For this pilot study, all non-communicative adult patients over the age of18 who were under deep sedation (defined by a RASS score less than or equal to −4) for organ failure [defined by at least an organ Sequential Organ Failure Assessment (SOFA) score (21) greater than or equal to 3], except for neurological failure, and who were expected to require 48 or more hours of ICU care were eligible for enrollment.

**Figure 1:**
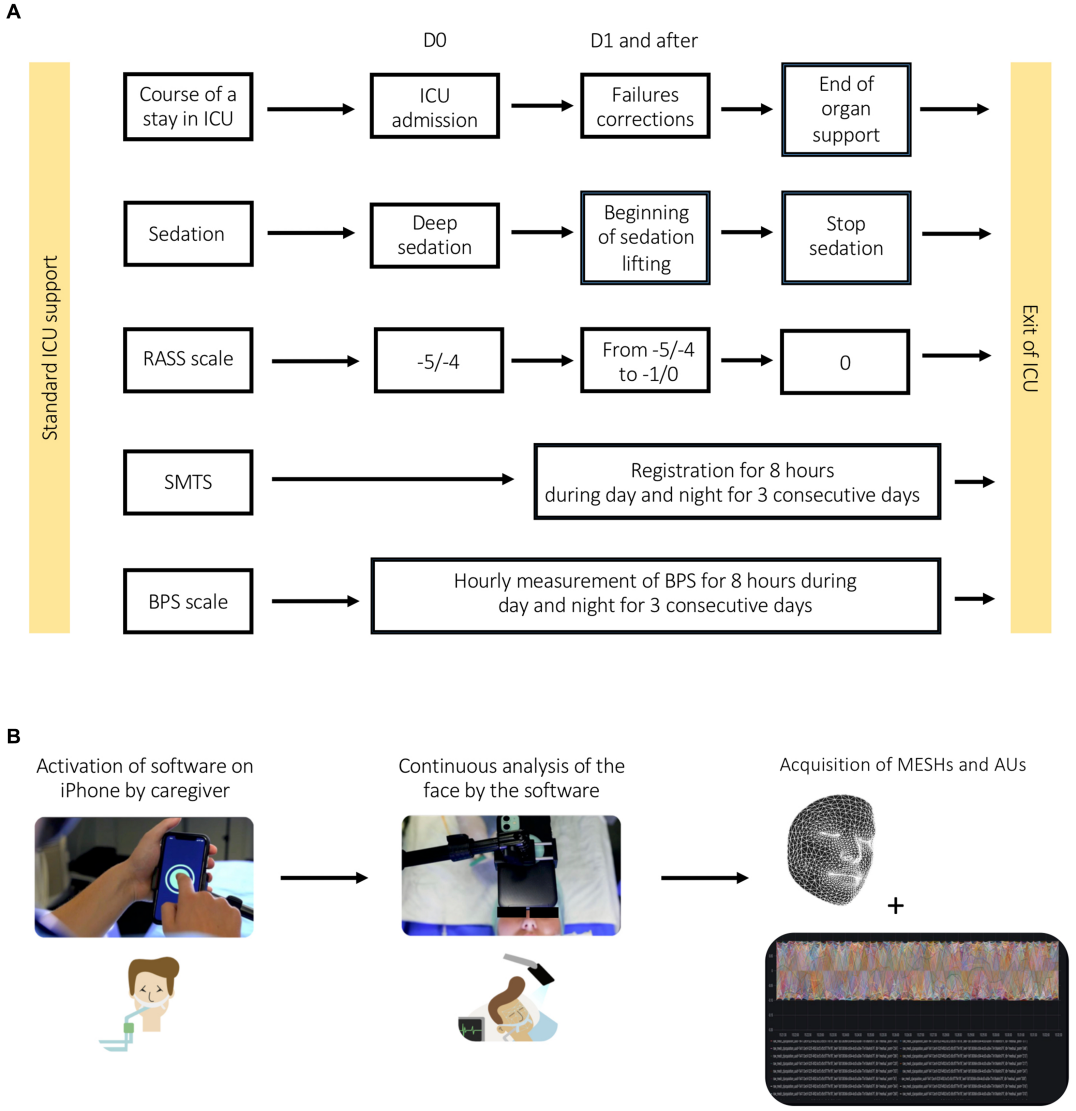
**(A)** Pilot study experimental plan. **(B)** Pipeline for data acquisition.

For each patient, the BPS [standard of care in our ICU and grading the facial expression-based pain score in accordance with the guidelines (9)] was collected hourly over two 8 h periods per day, diurnal and nocturnal, for 3 consecutive days (Figure 1A). iPhones were placed above the heads of patients admitted to the ICU and fulfilling the inclusion criteria (Figure 1B). Simultaneously, automated facial expression analysis by SMTS software was recorded continuously over the two 8 h periods per day for 3 days.

### 2.2 Characteristics of patients

For each patient, the Simplified Acute Physiology Score (SAPS II) (22) and the SOFA (21) were recorded within the first 24 h after ICU admission. Demographic and clinical data collected were as follows: age, sex, primary diagnosis at admission, and organ support during the study period such as mechanical ventilation, vasopressors, and renal replacement therapy. ICU length of stay before inclusion in the study, as well as mechanical ventilation duration, ICU length of stay, and ICU mortality, were also recorded. Regarding sedation-analgesia, the type and mean dose of each drug used during protocol timelapse, as well as RASS and BPS scores, were recorded according to the protocol.

### 2.3 Data description and image pre-processing

SMTS software is based on the analysis of patients’ facial expressions, coded in AUs using the FACS. The FACS system is used to measure human facial expressions metrically. The system was developed to describe facial movements, resulting from facial muscle activity in 46 AUs. Each AU code for a muscle or group of muscles was typically observed during the production of facial expressions under the influence of emotion. Four AU combinations have been described as including most of the pain-related information: AU4 (eyebrow lowering), AU6 + 7 (orbital tightening), AU9 + 10 (levator muscle contraction), and AU43 (eyes closed) (23, 24).

The data of interest in this clinical investigation is a mesh, which is a set of 1,220 points analyzed in four dimensions (three spatial and one temporal, at an average frequency between 14 and 45 Hz) defining the mesh of the patient’s facial expressions collected due to a sensor already present on the front digital camera of the hardware. iPhones (versions X, 11 and 12) were used to capture the patient’s 3D facial mesh, using the augmented reality library (ARKiT library), which in turn uses the iPhone’s red-green-blue (RGB) front camera. The facial mesh is acquired at an average speed of 20 meshes per second, but at variable speeds ranging from 15 to 45 meshes per second, depending on hardware and environmental conditions. The 1,220 points are placed in a 3D space, where the origin corresponds to a virtual point behind the face, and values are measured in meters. The positions of the points are invariant to the rotation and the distance of the head from the sensors; therefore, the movement of the points can only correspond to a change in the facial expression of the captured face.

**Figure 2:**
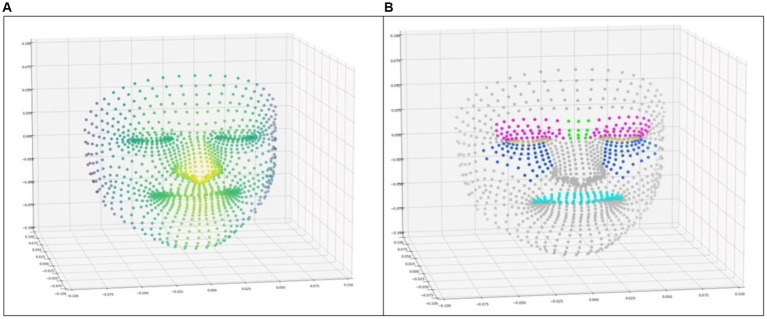
**(A)** Representation of the points of the facial mesh in 3 dimensions, with the color illustrating the depth. **(B)** Facial mesh points with the coloration of the points selected to represent some action units. (Pink: AU4; blue: AU6; yellow: AU7; green: AU9; and cyan: AU10).

These data were anonymized, analyzed, and converted into AU-related features as a function of time (Figure 2A).

### 2.4 Action unit features and data pre-processing

The features corresponding to AU4, AU6, AU7, AU9, and AU10 were implemented, but not AU43, which represents closed eyes, as ICU patients are expected to have their eyes closed most of the time. Each feature is calculated by applying one of the two calculation strategies to a defined subset of the 1,220 points in the facial mesh. The subset of points for each AU was chosen by hand to best match the AU (20) (Figure 2B).

The two computation strategies are:

- Strategy 1 (s1): Average distance between each point in the subset and a point on the top of the nose that is not expected to move (see Eq. 1). Strategy s1 is justified by the fact that it detects the movement of points toward the nose when the face frowns under the effect of pain.

(1) $$\mathrm{AU}\_XXs1=\frac{1}{n}\;\underset{p_{n}\in P}{\sum }\|p_{n}-p_{\mathrm{nose}}\|{\;}_{2}$$

Computation of strategy s1 for a given AU, where *P* is the set of 3D points associated with the AU, $p_{\mathrm{nose}}$ is a point chosen on the top of the nose, and *n* is the number of points in the set *P*.

- Strategy 2 (s2): Average distance between each point in the subset and the centroid point of the subset (see Eq. 2). The rationale behind strategy s2 is that it detects contraction movements in an area where each point of the subset gets closer to each other.

(2) $$\mathrm{AU}\_XXs2=\frac{1}{n}\;\underset{p_{n}\in P}{\sum }\|p_{n}-p_{\mathrm{centroid}}\|{\;}_{\;2}\;\mathrm{with}\;p_{\mathrm{centroid}}=\frac{1}{n}\underset{p_{n}\in P}{\sum }p_{n}$$

Computation of strategy s2 for a given AU, where *P* is the set of 3D points associated with the AU, $p_{\mathrm{centroid}}$ is the centroid point of the set *P*, and *n* is the number of points in the set *P*.

Features AU4s1, AU6s1, and AU10s1 were computed using strategy s1 and represent AU4, AU6, and AU10. Features AU4s2, AU7s2, and AU9s2 were computed using strategy s2, and represent AU4, AU7, and AU9. Two features are computed for AU4, AU4s1, and AU4s2, in order to better represent this AU, which can be seen as a combination of a movement of the eyebrows toward the nose and the contraction of the eyebrows toward each other. The calculation of AU7s2 is a little different, as it corresponds to the mean value of the values obtained by the s2 strategy to each eyelid separately. These characteristics do not predict the activation of an AU or its level of activation. Rather, they are supposed to correlate with the level of activation of each AU for a given individual but may be affected by morphological differences between individual patients.

Once the AU characteristics have been calculated, a certain amount of data pre-processing is required. Indeed, for environmental reasons, the ARKit library is not always able to correctly detect the patient’s face and calculate a facial mesh. For this reason, we find in our data not only noise affecting the position of points but also the complete absence of data during certain periods of time. Moreover, when ARKit redetects the face after the absence of data, a short mesh period given by the library corresponds to a readjustment of the mesh with the patient’s face, resulting in a poor-quality mesh with facial dimensions and points positions that do not match reality. Fortunately, the readjustment period lasts less than half a second. For these reasons, the following data pre-processing was applied:

- Remove data corresponding to the readjustment period by removing the first second of data following the absence of data of 0.1 s.
- Remove periods of uninterrupted data that are too small, lasting less than 20 s. We consider short periods of uninterrupted data to be unsafe, as they indicate that it is difficult to detect the face correctly over this period of data.
- Smooth out the noise by calculating a moving average of size 20 on the AUs features.

### 2.5 Primary objective (Calibration tool)

Model design and calibration of the innovative medical device (*NEVVA^©^*) for the automated, continuous and three-dimensional analysis of facial expressions. The fitted model will be evaluated mainly by the root mean square error (RMSE) between the BPS evaluated by the nursing staff and the BPS calculated by the algorithm.

### 2.6 Statistical analysis

Patient data are expressed as number (percentage) for categorical variables and as mean ± standard deviation (SD) for continuous variables.

All the data collected at the end of the study are grouped for a given patient at a rate of 20 measurements per second over 8 h of recording during the day and 8 h of recording at night, and this for 3 days: 8 × 60 × 60 ×2 × 3 × 20 = 172,800 measurements. This total number of measurements makes it possible to study variations of the system as a function of different clinical situations: at rest, during treatment, during a medical procedure, during a painful episode, etc. A sample of 30 patients leads to the collection of 5,184,000 measurements.

**Figure 3:**
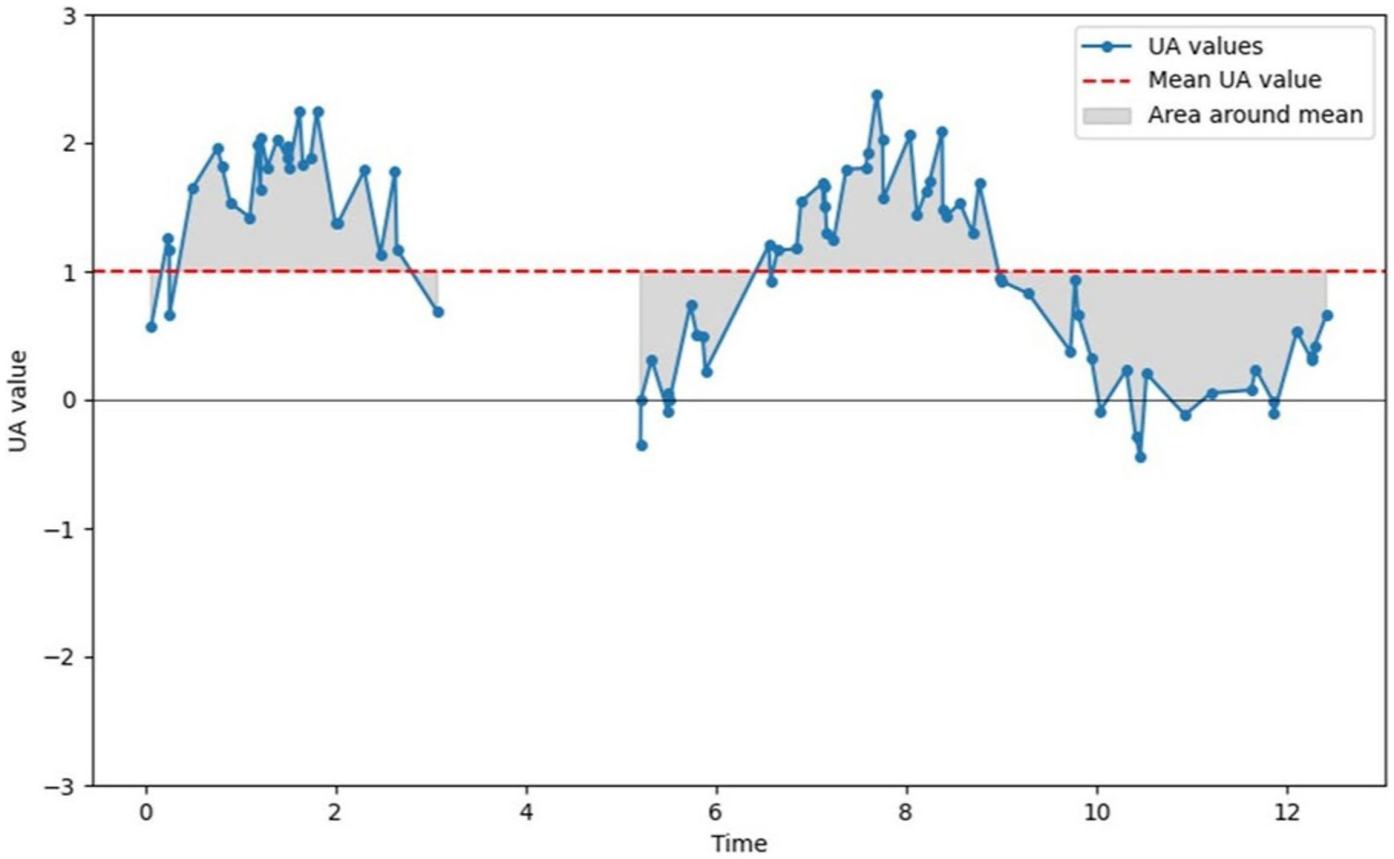
Illustration of the computation of the area around the mean statistic. The sum of all gray areas constitutes the “area around mean” statistic.

Given our objective to predict the presence or absence of pain over a given time interval, and in accordance with the pain recorded by healthcare staff, feature statistics are computed for a given time interval. The following statistics are calculated: mean, minimum, maximum, variance, SD, and area around the mean. The logic behind the unusual calculation of the area around the mean is that it measures both the duration of a series of movements and their intensity. To calculate this statistic, we first calculate the mean value of the characteristic in the time interval, subtract the mean value of the characteristic values in the time interval, and, finally, calculate the integral, using the trapezoidal rule, by adding up the absolute integral value of the parts that have only positive values and the parts that have only negative values. Figure 3 illustrates the computed statistic. To compensate for potential data absences, the statistic is finally divided by the sum of the uninterrupted data durations, and the zones associated with a data absence are not taken into account in the zone sum.

**Figure 4:**
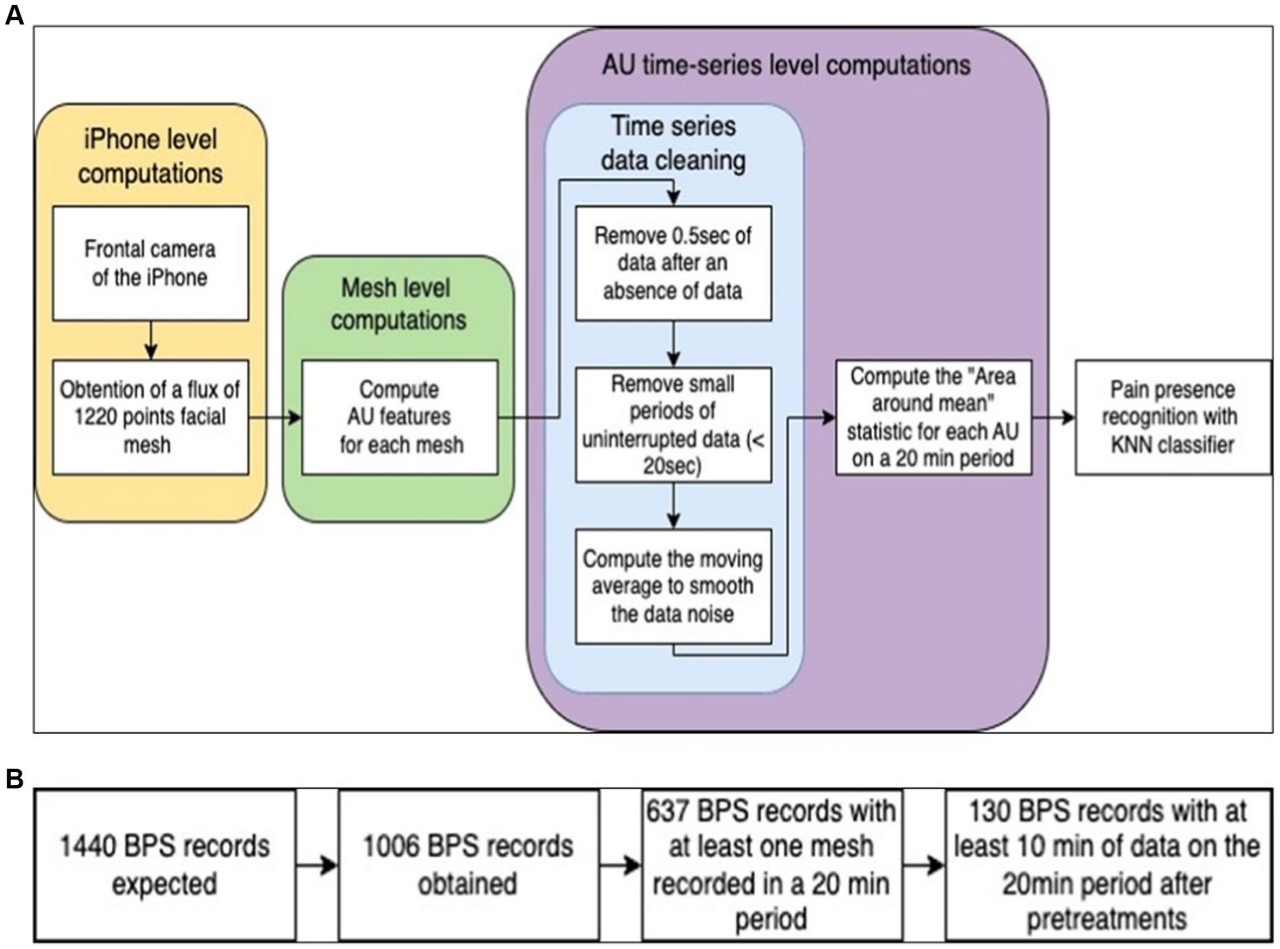
**(A)** Schematization of the proposed approach. **(B)** Schematization of the data selection process.

In essence, our approach involves first calculating the AU features for each mesh acquired in the time interval of interest, then applying the various pretreatments on the AU features time series and finally calculating all the statistics described for each feature on the given time interval, giving us a total of 36 variables (6 AU features × 6 statistical calculations) to describe a patient’s pain-related movements over a given time interval. Our approach is shown in Figure 4A.

We use Python v3.11.4 (Python Software Foundation, Beaverton, Oregon, United States) for statistical analyses.

### 2.7 Ethic issues

The NEWA study was approved by the Local Health Research Ethics Committee of Caen University Hospital (ID 2980), and all methods were performed in accordance with the relevant guidelines and French research laws. Written informed consent was obtained from patients and/or their surrogates prior to study enrolment and data collection. The procedures were carried out as part of the daily management of patients admitted to the medical ICU (analysis and recording of data for calibration of the device, without modification of overall management).

## 3 Results

### 3.1 Patients’ and BPS recordings’ characteristics

A total of 30 participants were enrolled between March and July 2022. The mean age of the included patients for analyses was 57 years, and 60% of them were male patients. SOFA and SAPS II were 9 and 47, respectively. All participants had an RASS score less than or equal to −4 at inclusion, 46% had a median RASS score from −4 to −3 on day 1 and 23% were not evaluable on RASS because they were awake and communicating. Thirty percent of patients had a median RASS score ranging from −4 to −3 on the second day and 50% could not be evaluated on RASS because they were awake and communicating (Table 1).

**Table 1:** Patients’ characteristics.

Characteristics and variables	All participants (*N* = 30)
Inclusion period	March 2022 to July 2022
Age, median (range)	57 (32–70)
Male, number (%)	18 (60)
Female, number (%)	12 (40)
Primary diagnosis at admission, number (%) Coma Respiratory failure Hemodynamic failure Cardiac arrest	5 (17) 20 (67) 3 (10) 2 (6)
Maximum SAPS II score, median (range)	47 (25–78)
Maximum SOFA score, median (range)	9 (2–13)
Any organ support, number (%)	30 (100)
Patients required vasopressors, number (%)	17 (57)
Patients required mechanical ventilation, number (%)	30 (100)
Patients required renal replacement therapy, number (%)	6 (20)
ICU length of stay before inclusion, median day (range)	2 (0–12)
ICU length of stay, median day (range)	12 (4–57)
Mechanical ventilation duration, median day (range)	7 (1–36)
Type of hypnotic at inclusion, number (%)—median dose (range) Propofol Midazolam Propofol + Midazolam Propofol + Ketamine Propofol + Dexmedetomidine	17 (57)–180 mg/H (100–250) 5 (17)–10 mg/H (5–30) 5 (17)–200 mg/H (100–300) + 10 mg/H (10–50) 1 (3)–300 mg/H + 150 mg/H 2 (7)–120 mg/H (80–150) + 0.7 𝜇g/kg/H (0.6– 0.7)
Sufentanil at inclusion, median dose (range)	10 𝜇*g*/H (3–20)
RASS score at inclusion, day +0 −5, number (%) −4, number (%) −3 at 0, number (%)	6 (20) 24 (80) 0 (0)
Median RASS score at day +1 −5, number (%) −4, number (%) −3, number (%) −2, number (%) −1, number (%) 0, number (%) NA, number (%)	0 (0) 7 (23) 7 (23) 3 (10) 3 (10) 3 (10) 7 (23)
Median RASS score at day +2 −5, number (%) −4, number (%) −3, number (%) −2, number (%) −1, number (%) 0, number (%) NA, number (%)	1 (3) 5 (17) 4 (13) 1 (3) 1 (3) 2 (7) 15 (50)
ICU mortality, number (%)	2 (7)

NA, not applicable; RASS, Richmond Agitation and Sedation Scale; SOFA, Sequential Organ Failure Assessment; SAPS, Simplified Acute Physiology Score.

For medical and care reasons, 1,006 of the 1,440 expected BPS recordings were obtained. For each recording, a time interval of 10 min before and 10 min after the recording was expected. Recordings containing no data during this 20 min period were immediately discarded. As a result, 637 BPS recordings were retained (Table 2).

**Table 2:** BPS records’ characteristics before and after treatment.

Facial expression and total BPS records			
	Quantity of records		Quantity of records
BPS facial expression 1	525	Total BPS 3	473
BPS facial expression 2	109	Total BPS 4	102
BPS facial expression 3	3	Total BPS 5	40
BPS facial expression 4	0	Total BPS 6	16
		Total BPS 7	5
		Total BPS 8	1
		Total BPS > 8	0

Facial expression BPS records after treatments	
	Quantity of records
Absence of pain	108
Presence of pain	22

Some dataset recordings lacked data for the 20 min study period. Recordings with a cumulative total of less than 10 min of data for the 20 min recording period were removed from the dataset. Finally, 130 recordings out of the 637 previously selected were retained for the final analysis (Figure 4B).

Data diversity across pain levels is poor, especially for high pain levels. For this reason, we focused our study on the ability to recognize the presence of pain rather than on the recognition of pain intensity. Next, 108 recordings of BPS facial expressions corresponding to an absence of pain (facial BPS = 1) and 22 recordings of BPS facial expressions recordings corresponding to the presence of pain (facial BPS ≥ 2) were obtained (Table 2).

### 3.2 Patient facial AU detection and model training

Numerous experiments were carried out, involving various types of machine learning models, hyperparameters fitting, and variable selection. A k-nearest-neighbor (KNN) classifier model, using the “area around mean” statistics of AU4s1 and AU4s2 as model inputs, produced the best results. This may be explained by the fact that eyebrow movement, represented by AU4, is the most common and visible movement when expressing pain.

### 3.3 Primary objective—Calibration tool

Due to the small size of the dataset, a leave-one-subject-out cross-validation (LOSO-CV) strategy was used. Prior to each training session, and to mitigate the effect of imbalanced data, reduced sampling of the training data was performed to get an equal number of painful and non-painful examples. KNN hyperparameters were tuned by testing a large set of possible combinations of hyperparameter values, with a LOSO-CV strategy and to maximize the F1-score of the classifiers produced.

**Figure 5:**
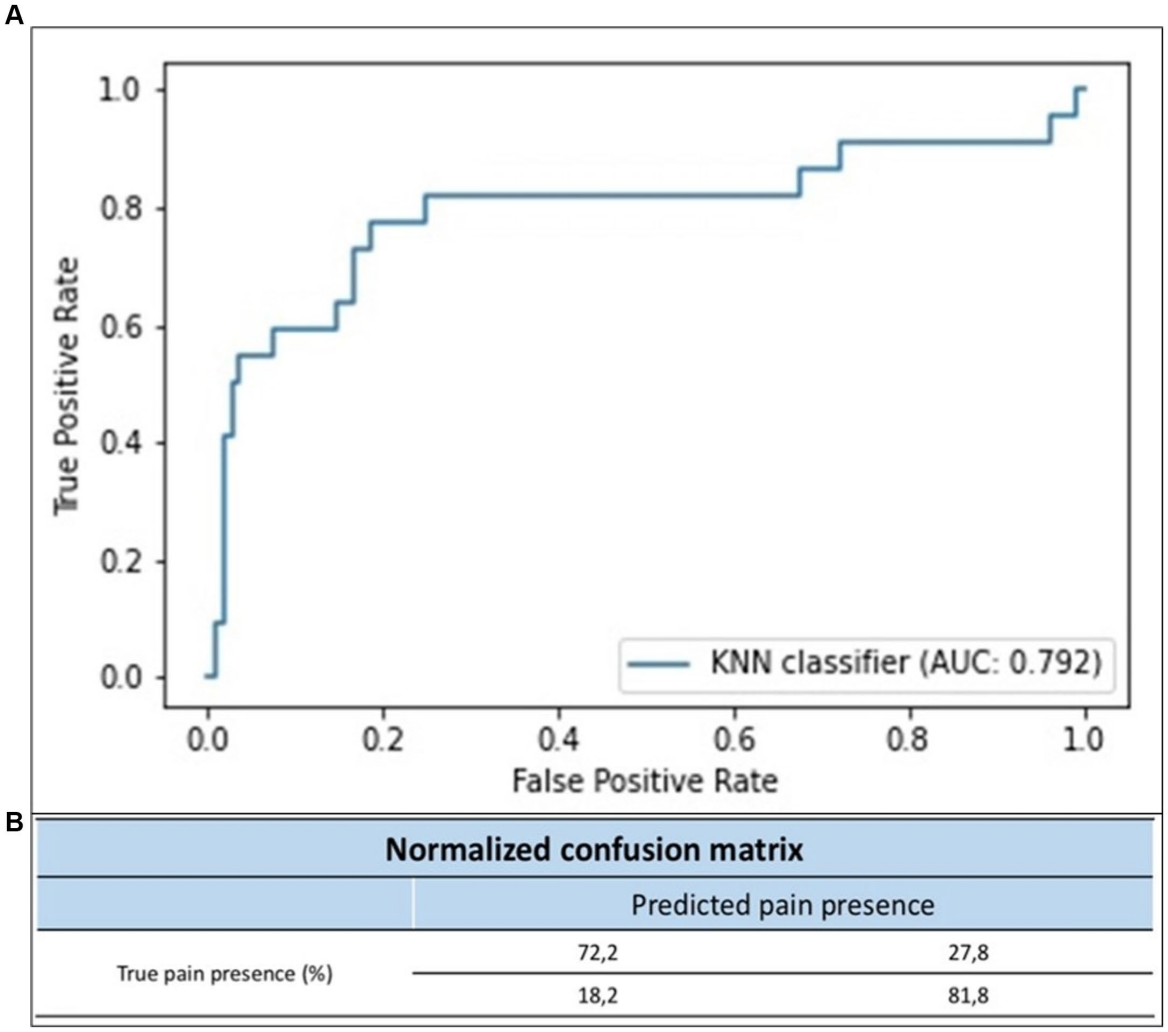
**(A)** ROC curve of the KNN classifier. **(B)** Normalized confusion matrix in proportion of each class.

With the presented model, dataset, and training approach, we obtained an area under the curve (AUC) with a 95% confidence interval of (0.735–0.803), using a bootstrapping method to calculate the AUC confidence interval, with 2000 stratified bootstrap replicates. As an example, the results of a LOSO-CV-adjusted classifier on the non-bootstrapped dataset can produce a receiver operating characteristic (ROC) curve with an AUC of 0.792 (Figure 5A). By adjusting the classifier’s decision threshold to our preferences, we obtain a model with a sensitivity of 0.818 and a specificity of 0.722 (Figure 5B).

## 4 Discussion

In this prospective pilot study, we set up a protocol for applying AI to obtain facial expression analysis in critically ill, non-communicative patients. We focused on a facial mesh composed of 1,220 points analyzed in four dimensions, defining the patient’s facial expressions. Using FACS to measure these human facial expressions metrically using AUs, we have established a pain classification based on facial expression. Meshes were collected using a sensor present on an iPhone and a digital camera. The device’s performance in detecting pain was close to 80% in sensitivity and 70% in specificity. The present study demonstrates the feasibility of automated, continuous pain assessment using a novel AI tool in ICUs.

Optimal pain management in ICU patients poses several challenges, such as the lack of clearly defined management protocols for certain painful conditions, the fear of adverse effects from analgesic drugs, uncertainty regarding the reliability and specificity of certain behavioral indicators of pain, and limited accuracy in interpreting facial expressions associated with negative effects. The application of AI for automated facial analysis is a dynamic area of human emotion research, with many commercial software tools available for automated facial coding. Some of these tools [e.g., Noldus FaceReader (25) and Affdex (26)] can automatically detect facial AUs in accordance with FACS. Automated tools are often considered to offer greater objectivity and reliability than human assessment, as they can eliminate subjectivity and bias (27).

In the context of pain assessment, a few studies have focused on the evaluation of facial expression in humans, particularly in infants. The Prkachin and Solomon Pain Intensity score is a valuable tool for assessing pain intensity based on FACS AUs (28). Zamzmi et al. (29) reviewed the most recent methods of automated pain analysis in infants, and facial expressions are considered one of the most common and specific indicators of pain. The facial expression of pain involves movements and distortions in facial muscles associated with a painful stimulus, including deepening of the nasolabial furrow, brow lowering, narrowed eyes, and chin quiver. Many important aspects of patient care are not yet captured autonomously. AI is developing rapidly in the medical field, and its scope of application is vast in the ICU setting. AI technology could help not only to perform repetitive assessments in real-time but also to integrate and interpret these data sources in relation to electronic medical record data, potentially enabling more timely and targeted interventions (30, 31). For example, non-invasive monitoring of ICU patients and their environment with an AI system has recently been shown to be feasible and effective in differentiating the behavior of patients with or without delirium (32).

Sensitive and reliable pain assessment is difficult to achieve for ICU patients under deep sedation who are unable to self-report their pain. Facial responses to pain appear to be consistent across distinct types of pain stimulation (33). The use of AI-based interventions in conscious patients has a positive effect on pain recognition, pain prediction, and pain self-management. Most reports, however, are only pilot studies (34). Using imaging analysis, Kuramoto et al. (35) explored the physiological basis of how pain signaling leads to pain-indicative muscle movement in 18 healthy patients. They used iPhone sensors to acquire facial meshes and reported AI-based analyses focusing on the facial area. Our study confirms the accuracy of this kind of AI tool for critically ill, non-communicative patients.

In the ICU, it is estimated that over 50% of patients experience moderate to severe pain at rest, while 80% of patients experience pain during procedures. Over the past 2 decades, pain assessment has been improved by the widespread use of pain scales, such as BPS and CPOT, in which analysis of facial expression is the main factor. Nevertheless, under appropriate sedation, in the most severe patients, pain-induced changes in facial expression are difficult to identify (36). Recently, Wu et al. (37) produced a video-based pain classification for ICU patients, which yielded a sensitivity of 0.802 for detecting grimacing versus relaxed facial expression. In our study, using our *NEVVA^©^*-generated database and with a LOSO-CV strategy, we fitted a model that yielded an AUC of 0.792, a sensitivity of 0.82, and a specificity of 0.72.

AI-based methodologies can streamline pain prediction, recognition, and scoring processes and contribute to the automatic identification of pain from clinical notes containing pertinent pain assessment data. The interest of our methodological, specific approach is also reflected in the stepwise calibration on a decreasing sedation scale from RASS score (−5/−4 at the start of inclusions to awakening) and day/night continuity in order to study the overall changes in automated facial analysis as a function of sedation regime and circadian rhythm. Although *NEVVA^©^* could be a valuable tool for assessing pain in sedated patients, certain methodological remarks must be highlighted in the present study. First, to minimize the inclusion of patients with compromised neurological status, we excluded patients with primary neurological failure as well as those receiving neuromuscular blockers; and second, analysis by the *NEVVA^©^* system is equated with continuous scales (e.g., visual analog scale), which are temporally more relevant than categorical scales (e.g., BPS) for assessing pain, to establish linearity in the recording of pain intensity (38).

In critically ill patients, regular pain assessment is associated with a better outcome, as are ventilator-free days. On the one hand, severe pain may reflect the potential deterioration of serious illness, but on the other hand, increasing pain has been associated with anxiety, delirium, and poor short- and long-term outcomes (39). The results obtained in this pilot study are encouraging for future research. AI-based automated pain assessment could be used in the future as a continuous monitoring tool to indicate the need for immediate assessment and management by nursing staff. This low-cost, high-capacity, intelligent data processing could also enable earlier identification of the onset of pain and ensure ongoing monitoring, thus better distributing nurses’ workload so that they can devote time to their core tasks.

However, our study has several limitations, the main one being that the tool was only able to identify the presence or absence of pain signals but not discern the specific characteristics and severity of pain. This underscores the need for further investigation in this area to develop more nuanced and accurate pain assessment techniques. Second, this is a single-center study, but the management of pain is in line with international guidelines. Third, the model used focuses on facial expression, but in the absence of brain injury or neuromuscular blocking agents, we assume that changes in facial expression are most relevant to assessing pain in critically ill patients. For example, vital signs often fluctuate without precision during nociceptive procedures in the ICU (40), but facial muscle movements associated with pain may be weak due to tissue edema (inducing a loss of dynamic change of muscle movement AUs). In addition, the quality of the results was hindered by the fact that a large number of records were not exploitable, mainly due to an insufficient amount of mesh data acquired at the BPS record time, which led to having only a small dataset to analyze. This lack of data was explained by an improper placement of the iPhone toward the face of the patient and by insufficient room lighting. Moreover, the proposed model is also limited by the large time interval needed to predict the presence or absence of pain. We were unable to reduce the size of the time interval without adversely affecting the results. One explanation is that BPS recording times were noted on paper and may therefore generate temporal inaccuracies. Given that painful expressions can evolve in a matter of minutes, it is possible that by having a small analysis time interval, we associate non-painful expressions with a painful BPS recording, or inversely. However, with a large time interval, the painful expression would be present in the analyzed data, even with a large time gap with the recording. In order to improve our model, a second stage of this study is underway in our ICU to tackle these latter issues. An iPad-based interactive software application has been developed for BPS data capture to replace paper recording. This software accurately records the BPS recording time with timestamps and checks that the iPhone mesh acquisition is working correctly so that BPS and iPhone recordings are obtained at the same time. In the event of an acquisition problem, staff are informed by a color code and asked to check the iPhone’s positioning and the room brightness before starting BPS recording. Due to this technological upgrade, a higher-quality dataset should increase the operability and performance (i.e., sensibility and specificity) of our AI model. Additionally, *NEVVA^©^* could be used in decision-making processes to measure the efficacy of analgesia and determine the impact of analgesia titration on patient outcomes, such as the duration of mechanical ventilation and length of ICU stay. Future research should incorporate controlled trials to assess the effectiveness of these innovative systems in improving pain management.

## 5 Conclusion

In 1872, Darwin explained how different affective states, including pain, manifest themselves through distinct behaviors, including facial expressions (41). One hundred and forty years later, autonomous pain assessment based on facial expression is a key issue for critically ill patients but is somewhat difficult to assess in the ICUs due to the lack of communication among patients under deep, multimodal sedation. In the present prospective study, we developed and calibrated an innovative medical device—*NEVVA^©^*—an automated pain assessment tool based on facial expression in critically ill patients with good sensibility and specificity.

These findings enable AI-based pain assessment in ICUs by monitoring changes in facial expressions in critically ill patients. However, further studies are warranted to validate the performance of this new automated pain assessment tool.

## Data availability statement

The original contributions presented in the study are included in the article/supplementary material, further inquiries can be directed to the corresponding author.

## Ethics statement

The studies involving humans were approved by Health Research Ethics Committee of the University Hospital of Caen. The studies were conducted in accordance with the local legislation and institutional requirements. The participants provided their written informed consent to participate in this study.

## Author contributions

MB: Writing – review & editing, Writing – original draft, Visualization, Validation, Supervision, Software, Resources, Project administration, Methodology, Investigation, Funding acquisition, Formal analysis, Data curation, Conceptualization. JL: Writing – review & editing, Writing – original draft, Visualization, Validation, Supervision, Software, Resources, Project administration, Methodology, Investigation, Formal analysis, Data curation, Conceptualization. TC: Writing – review & editing, Writing – original draft, Visualization, Validation, Supervision, Software, Resources, Project administration, Methodology, Investigation, Funding acquisition, Formal analysis, Data curation, Conceptualization. AH: Writing – review & editing, Supervision, Software, Resources, Project administration, Methodology, Investigation, Funding acquisition, Formal analysis, Data curation, Conceptualization. TG: Writing – review & editing, Resources, Project administration, Methodology, Investigation, Formal analysis, Conceptualization. PB: Writing – review & editing, Visualization, Resources. PC: Writing – review & editing, Visualization, Resources. CDa: Writing – review & editing, Visualization, Resources. CDe: Writing – review & editing, Visualization, Resources. AD: Writing – review & editing, Visualization, Resources. SG: Writing – review & editing, Visualization, Resources. AJ: Writing – review & editing, Visualization, Resources. MM: Writing – review & editing, Visualization, Resources. XV: Writing – review & editing, Visualization, Resources. DCe: Writing – original draft, Visualization, Project administration, Methodology, Formal analysis, Data curation, Conceptualization. RM: Writing – original draft, Visualization, Validation, Project administration, Methodology, Formal analysis, Data curation, Conceptualization. PM: Writing – original draft, Visualization, Project administration, Methodology, Formal analysis. FC: Writing – original draft, Supervision, Project administration, Methodology, Investigation, Formal analysis, Conceptualization. JD: Writing – review & editing, Visualization, Project administration, Methodology, Investigation, Formal analysis, Conceptualization. DV: Writing – original draft, Validation, Project administration, Methodology, Investigation, Conceptualization. DCh: Writing – review & editing, Visualization, Validation, Supervision, Software, Resources, Project administration, Methodology, Investigation, Formal analysis, Conceptualization.

## Appendix

**Table A1:** Richmond Agitation-Sedation Scale.

Richmond Agitation-Sedation Scale (RASS)		
Score	Term	Description
+4	Combative	Overtly combative, violent, immediate danger to staff
+3	Very agitated	Pulls or removes tube(s) or catheter(s), aggressive
+2	Agitated	Frequent nonpurposeful movement, fights ventilator
+1	Restless	Anxious but movements not aggressively vigorous
0	Alert and calm	
−1	Drowsy	Not fully alert but has sustained awakening to voice (≥10 s)
−2	Light sedation	Briefly awakens to voice with eye contact (<10 s)
−3	Moderate sedation	Movement or eye opening to voice (but no eye contact)
−4	Deep sedation	No response to voice but movement or eye opening to physical stimulation
−5	Unarousable	No response to voice or physical stimulation

**Table A2:** Behavior Pain Scale.

Behavior Pain Scale (BPS)		
Sub-scale	Description	Score
Facial expression	Relaxed	1
	Partially tightened	2
	Fully tightened	3
	Grimacing	4
Movement of the upper limbs	No movement	1
	Partially bent	2
	Fully bent with finger flexion	3
	Permanently retracted	4
Compliance with mechanical ventilation	Tolerating movement	1
	Coughing but tolerating ventilation for most of the time	2
	Fighting ventilator	3
	Unable to control ventilation	4

Total 3 (No) to 12 (Maximum): Score 3 to 4 = No pain, Score 5 to 6 = Mild pain, Score ≥ 7 = Maximum pain.
